# Frequency and methylation status of selected retrotransposition competent L1 loci in amyotrophic lateral sclerosis

**DOI:** 10.1186/s13041-020-00694-2

**Published:** 2020-11-13

**Authors:** Abigail L. Savage, Ana Illera Lopez, Alfredo Iacoangeli, Vivien J. Bubb, Bradley Smith, Claire Troakes, Nada Alahmady, Sulev Koks, Gerald G. Schumann, Ammar Al-Chalabi, John P. Quinn

**Affiliations:** 1 Department of Pharmacology and Therapeutics, Institute of Systems, Molecular and Integrative Biology, University of Liverpool, Liverpool, UK; 2 Maurice Wohl Clinical Neuroscience Institute, King’s College London, London, UK; 3 Department of Biostatistics and Health Informatics, King’s College London, London, UK; 4 London Neurodegenerative Diseases Brain Bank, Institute of Psychiatry, Psychology and Neuroscience, Kings College London, London, UK; 5 Department of Biology, Imam Abdulrahman Bin Faisal University, Dammam, Saudi Arabia; 6 Perron Institute for Neurological and Translational Science, Perth, WA Australia; 7 Centre for Molecular Medicine and Innovative Therapeutics, Murdoch University, Perth, WA Australia; 8 Division of Medical Biotechnology, Paul-Ehrlich-Institut, Langen, Germany

**Keywords:** Amyotrophic lateral sclerosis, LINE-1, Retrotransposition competent, Methylation

## Abstract

Long interspersed element-1 (LINE-1/L1) is the only autonomous transposable element in the human genome that currently mobilises in both germline and somatic tissues. Recent studies have identified correlations between altered retrotransposon expression and the fatal neurodegenerative disease amyotrophic lateral sclerosis (ALS) in a subset of patients. The risk of an individual developing ALS is dependent on an interaction of genetic variants and subsequent modifiers during life. These modifiers could include environmental factors, which can lead to epigenetic and genomic changes, such as somatic mutations, occurring in the neuronal cells that degenerate as the disease develops. There are more than 1 million L1 copies in the human genome today, but only 80–100 L1 loci in the reference genome are considered to be retrotransposition-competent (RC) and an even smaller number of these RC-L1s loci are highly active. We hypothesise that RC-L1s could affect normal cellular function through their mutagenic potential conferred by their ability to retrotranspose in neuronal cells and through DNA damage caused by the endonuclease activity of the L1-encoded ORF2 protein. To investigate whether either an increase in the genomic burden of RC-L1s or epigenetic changes to RC-L1s altering their expression, could play a role in disease development, we chose a set of seven well characterised genomic RC-L1 loci that were reported earlier to be highly active in a cellular L1 retrotransposition reporter assay or serve as major source elements for germline and/or somatic retrotransposition events. Analysis of the insertion allele frequency of five polymorphic RC-L1s, out of the set of seven, for their presence or absence, did not identify an increased number individually or when combined in individuals with the disease. However, we did identify reduced levels of methylation of RC-L1s in the motor cortex of those individuals with both familial and sporadic ALS compared to control brains. The changes to the regulation of the loci encompassing these RC-L1s demonstrated tissue specificity and could be related to the disease process.

## Introduction

Long interspersed element-1 (LINE-1/L1) represents the only autonomous retrotransposon family in the human genome whose members are currently mobilised and it constitutes a significant source of endogenous mutagenesis [1]. L1 elements are non-long terminal repeat (non-LTR) retrotransposons, which propagate through a ‘copy and paste’ mechanism including L1 cDNA synthesis by a process termed target primed reverse transcription [2, 3]. A functional, full length L1 element is ~ 6 kb in length, contains both a 5′ and 3′-untranslated region (UTR), three open reading frames (ORF0, ORF1, ORF2) and a poly A tail at its 3′end, and is flanked by variable target site duplications. ORF1p, a ~ 40 kDa protein with RNA binding and chaperone activities, and ORF2p, a ~ 150 kDa protein with endonuclease and reverse transcriptase activities, are essential for L1 retrotransposition [4–7]. L1 sequences contribute to approximately 17% of the human genome with over one million copies, however, by far the majority are unable to mobilise due to 5′-truncations, internal deletions or rearrangements and mutations in the ORFs encoding the proteins required for retrotransposition [8, 9]. The GRCh38 release of the human genome contains 146 full-length L1 elements harbouring intact open reading frames (ORFs) [10]. It is estimated there are approximately 80–100 retrotransposition competent L1 loci (RC-L1) in the average human genome, however testing these elements in retrotransposition reporter assays in cell culture revealed that there are only six highly active or ‘hot’ L1 elements in the reference genome that were responsible for the majority of retrotransposition activity measured [11]. Approximately 15% of all L1 retrotransposition events in the human genome result in the transduction of 3′ flanking genomic sequences as the L1 encoded canonical polyadenylation signal is bypassed by RNA-Polymerase II allowing the new L1 insertions to be traced back to their source locus [12, 13]. This has identified highly active genomic RC-L1 loci that are responsible for many new insertions in the germline, in somatic cells or both [14–17]. In addition, many of these highly active RC-L1s are polymorphic for their presence or absence in the genome, therefore every individual has a different complement of RC-L1 loci, which could result in distinct amounts of functional L1 mRNA.

L1 retrotransposition is repressed by multiple cellular mechanisms including the methylation of a CpG island located in the 5′UTR acting to reduce the levels of L1 mRNA expressed [18–21]. However, L1 retrotransposition does occur in the developing embryo and in germ, tumour and neuronal cells generating either heritable insertions or somatic insertions that are present in only a specific cellular lineage or even a single cell [21–24]. The extent to which these new L1 insertions affect function will depend on their genomic integration sites and will range from loss of function mutations (when insertions disrupt exons) to changes in transcript levels of an expressed gene [25]. L1 insertions in both germ cells and embryonic stem cells have been shown to be the cause of genetic disease and the somatic retrotransposition of a particularly active RC-L1 copy into the *APC* gene initiated a case of colorectal cancer [26–28]. Somatic L1 retrotransposition occurs in the human brain with rates reported ranging from 0.04 to 13.7 L1 insertions per neuron, although there is much debate on the actual mobilisation frequency in this cell type [29]. It is hypothesised that a controlled level of somatic retrotransposition in neuronal genomes may contribute to neuronal plasticity however beyond a beneficial level it could be involved in neurodegeneration and disease [30].

Amyotrophic lateral sclerosis (ALS) is a neurodegenerative disease with an uncertain aetiology involving the rapid and progressive degeneration of motor neurons of the brain and spinal cord. It is usually fatal within 3–5 years of disease onset and there are no current treatments to reverse or stop the course of the disease [31]. ALS cases are often divided into those with a family history of the disease (familial, FALS) and those without (sporadic, SALS). ALS is thought to represent one end of a spectrum disorder with frontotemporal dementia (FTD) at the other end due to significant overlap of the genetics and altered cellular pathways involved in both diseases [32]. Protein aggregates of TAR DNA binding protein (TDP-43) are a hallmark of the majority of ALS cases and 45–60% of FTD cases and multiple studies have demonstrated that retrotransposons, including L1, are regulated by TDP-43 [33–35]. Several studies have also shown correlations between altered retrotransposon expression and ALS and FTD when comparing expression in the brain of individuals with and without disease and in animal models [36–40]. A recent study using post-mortem cortex samples identified three distinct subsets of ALS patients one of which was characterised by de-silencing of multiple families of transposable elements that can be assigned to the groups of both LTR and non-LTR retrotransposons (including L1) and associated with TDP-43 dysfunction (20% of patients) [36]. In other studies, retrotransposon expression was significantly altered in ALS patients who were positive for the hexanucleotide repeat expansion in the *C9orf72* gene but not in those who were negative for the expansion [39, 40]. In rodent and *Drosophila* models of TDP-43 pathology, TDP-43 represses non-LTR and LTR retrotransposon transcripts [37, 38]. Moreover a reduction in the binding of TDP-43 to retrotransposon transcripts was observed in the brains of FTD patients compared to controls [38]. The loss of nuclear TDP-43 in brain tissue from FTD-ALS patients has been associated with decondensation of chromatin flanking L1 loci and an increase in L1 DNA content [41]. In addition, a cellular model of TDP-43 loss resulted in an increase in L1 retrotransposition, which could be inhibited by antiretroviral drugs [41].

We hypothesise that either an increase in the genomic burden of RC-L1s could contribute to the genetic risk of an individual to ALS or epigenetic changes to these L1 loci altering their expression or both could play a role in disease development. To address these hypotheses, we generated a list of seven genomic RC-L1 loci (Table 1) that were reported to exhibit high retrotransposition frequencies in L1 retrotransposition reporter assays or were a source of a high number of L1 insertions in either the germline, somatic tissues or both to be the focus of this study [11, 14, 15, 17, 42]. Genotyping of specific polymorphic RC-L1 loci was performed on samples from the MNDA UK (Motor Neurone Disease Association United Kingdom) DNA bank cohort [43] and the UK Project MinE samples using PCR and tagging single nucleotide polymorphisms (SNPs), respectively [44, 45]. Genotypes of tagging SNPs served as a proxy for the specific RC-L1 genotype. In order to assess the relative methylation status of the specific RC-L1 loci shortlisted, we enriched methylated gDNA employing a methyl-binding domain (MBD) protein to produce methylated and unmethylated DNA fractions from cerebellum, motor cortex and blood of healthy controls and individuals with FALS and SALS. Genotyping of these selected RC-L1 insertions did not reveal any association with disease risk or an increased burden of their presence in individuals with ALS in the cohorts used in this study. However, methylation analysis of these elements in the brain identified a higher level of methylation in control individuals compared to those with familial or sporadic ALS, specifically in the motor cortex.

**Table 1:** Retrotransposition competent L1 elements chosen for genotyping and methylation analysis in ALS cohort

Name	Percentage of retrotransposition activity of L1RP [11, 14, 42]	Ref/non-ref	Insertion Allele Frequency [11, 15]	Number of germline offspring elements from 3′ transduction analysis [15]	Number of 3′ transduction somatic insertions in cancer [17]	Chromosomal loci
L1_chr2_q24.1	150	Non-ref RIP	na,0.16	41/121	21/655	chr2:156,527,848 intergenic
L1_chr6_q24.1	141	Non-ref RIP	na,0.18	14/121	98/655	chr6:13,191,033 Intron PHACTR1
L1_chrX_p22.2	132	Ref RIP	0.34,0.74	1/121	20/655	chrX:11,953,208 intergenic
L1_chr6_p22.3	112.7	Ref RIP	0.30,0.61	2/121	2/655	chr6:24,811,907 Intron FAM65B
L1_chr8_q24.22	89.4	Ref RIP	0.44,1	2/121	4/655	chr8:135,082,987 intergenic
L1_chr1_p12	–	Ref	1,1	13/121	7/655	chr1:119,394,974 intergenic
L1_chr22_q12.1	13.8	Ref	1,1	2/121	137/655	chr22:29,059,272 Intron TTC28

The RC-L1 elements were shortlisted based on their high level of activity in a cellular retrotransposition assay [11, 14, 42], high number of germline offspring elements from 3′ transductions analysis [15] or high number of somatic insertions in cancer from 3′ transductions analysis [17]. Non-ref RIP: L1s that are not in the human reference genome and are polymorphic for their presence/absence. Ref RIP: L1s that are present in the human reference genome and are polymorphic for their presence/absence. Ref: L1s present in the human reference genome and there is currently no evidence that they are polymorphic for their presence/absence

## Methods

### Genomic DNA samples

Genomic DNA (gDNA) samples for genotyping of the five specific polymorphic RC-L1 insertions were obtained from the MNDA UK DNA bank cohort (ref DNA0042). gDNA of 2 control, 2 familial ALS and 2 sporadic ALS cases was obtained from the cerebellum and motor cortex from the London Neurodegenerative Diseases Brain Bank. Of the two FALS cases one carried the SOD1 p.D101G variant and the other the TARDBP p.M337V variant. The 3 sporadic ALS gDNA samples from the blood and motor cortex were obtained from the Sheffield Brain Tissue bank. Sample details are summarised in Additional file 1.

### Genotyping of five polymorphic RC-L1 loci in the MNDA UK DNA bank and UK Project MinE cohorts

The five selected polymorphic RC-L1 insertions were genotyped in gDNA samples from the MNDA UK DNA bank cohort, London Neurodegenerative Diseases Brain Bank and Sheffield Brain Tissue bank using GoTaq hot start polymerase (Promega) under standard conditions. PCR assays to test for presence/absence of each RC-L1 locus were designed with three primers each, two flanking the insertion and one located in the L1 5′ UTR (5′AACTCCCTGACCCCTTGC 3′; position 206–223 of the benchmark L1.3 element (accession no: L19088.1) [46], enabling amplification of the empty site and/or the L1 5′ junction within the same reaction (Fig. 2d). For two of the RC-L1 loci this PCR design was not appropriate due to differences in conditions required for each primer set. Therefore two PCR reactions were carried out, one for the empty site and another for the L1 5′ junction. Primer sequences are listed in Additional file 2. DNA samples in the MNDA UK DNA bank cohort have been sequenced as part of Project MinE. Therefore, SNP genotype data was available and the genotypes of candidate tagging SNPs for each of the polymorphic RC-L1s were obtained. Linkage disequilibrium (LD) between the SNPs and RC-L1 loci for those samples that both genotypes were available for, was calculated using PLINK (v1.07) [47]. The genotypes of the five polymorphic RC-L1 loci were then determined by the genotypes of the proxy SNPs in the Project MinE UK samples. Association analysis of the RC-L1 insertions and ALS was carried out using a Chi-squared test (PLINK v1.07). Two-sample test for equality of proportions with continuity correction combined with Chi-squared test was used to evaluate the proportions of insertion numbers of RC-L1 between cases and controls. Logistic regression was performed to determine if the total number of RC-L1s present at the 5 polymorphic loci were associated with disease status.

### Isolation of methylated and unmethylated DNA

Genomic DNA (gDNA) from controls, familial and sporadic ALS patients was sheared using a S220 focused-ultrasonicator (Covaris) with the following parameters duty cycle 5%, intensity 3 and cycles per burst 200 for 40 s × 4 to obtain fragments of 500 bp. The sheared gDNA was then purified and concentrated using a 1:1.1 ratio of DNA to Agencourt AMPure XP Beads (Beckman Coulter). Methylated and unmethylated DNA was isolated from 300 ng of each gDNA sample using the CpG MethylQuest kit (Millipore) according to manufacturer’s instructions. The CpG island of *SNRPN*, an imprinted locus, was used as positive control for the successful enrichment of gDNA in both methylated and unmethylated fractions and to determine if there was relative equal representation of methylated and unmethylated alleles in the respective fractions of gDNA. The volume of isolated gDNA from the methylated and unmethylated fractions that yielded equal signal intensities in the PCR for the *SNRPN* locus was used as input for the amplification of the 5′ junction of the RC-L1s and the empty sites.

### Quantification of the methylation status of CpG islands of selected RC-L1 loci

The 5′ junctions of 6 RC-L1 loci from Table 1 (except L1_chr2_q24.1) and the *SNRPN* locus were amplified in the methylated and unmethylated fractions of gDNA using primers in Additional file 2 and GoTaq hot start polymerase (Promega) under standard conditions. L1_chr2_q24.1 could not be amplified in the fragmented gDNA, as a large PCR product (985 bp) was required due to the presence of the highly repetitive region in which the L1 has inserted. Not including L1_chr2_q24.1 the length of flanking nucelotide sequence included in the PCR product of the RC-L1 5′ junction was 158-447 bp. The polymorphic RC-L1s had been genotyped in the gDNA of the individuals examined. Therefore, in those individuals who were heterozygous for the presence of the RC-L1, the empty site was also amplified to compare the methylation status of the allele lacking the insertion. The PCR products were separated using gel electrophoresis, and the intensities of the bands were measured using Image J software (the software of the transilluminator (Biorad Molecular Imager, Chemidoc XRS +) highlighted saturated signals of PCR products to ensure the images used in downstream analysis were not including saturated signals). The relative intensity of the PCR products from the methylated and unmethylated fractions of DNA from the same individual were compared to determine the percentage which was methylated. A two-tailed student’s T-test was used to analyse the statistical differences between the proportion of the RC-L1s that were methylated between different tissues and disease states.

## Results

### Investigating a potential association of five specific, highly active polymorphic RC-L1 loci with ALS

Seven genomic RC-L1 loci were selected from the literature due to their exceptionally high retrotransposition rate (89.4 to 150% of L1_RP_) as determined in L1 retrotransposition reporter assays in cell culture [11, 14, 42] or because they were shown to serve as highly active source elements in vivo in the germline or in tumours [15, 17] (Table 1). The L1 in vivo activity of individual RC-L1 source loci had been quantified by the mapping of 3′ transduction events of de novo L1 insertions that were derived from these source loci (Table 1) in the germline [15] or in tumour tissues [17]. The seven RC-L1 loci were chosen as they were referred to as ‘hot’ or exceptionally active in at least one of the parameters outlined above. In Gardner et al. 38 RC-L1 loci were identified as generating germline 3′ transduction events and the three loci (L1_chr2_q24.1, L1_chr6_q24.1 and L1_chr1_p12) gave rise to more than half of these transductions (Table 1) [15]. In Tubio et al. the two ‘hot’ RC-L1_loci chr6_q24.1 and L1_chr22_q12.1 together were responsible for more than a third of the somatic transduction events (Table 1) [17]. Finally Brouha et al. defined ‘hot’ L1s as those elements that demonstrated a third of the activity of a known highly active element (L1_RP)_) in a cellular retrotransposition assay and L1_chrX_p22.2, L1_chr6_p22.3 and L1_chr8_q24.22 showed the three highest percentage activities of 82 elements tested [11]. With the exception of the two highly active non-reference RC-L1 insertions L1_chr2_q24.1 and L1_chr6_q24.1 (Table 1), which are polymorphic for their presence/absence and are not part of the reference genome, the remaining five RC-L1 loci are annotated in the hg19 reference genome, three of which are also polymorphic for their presence/absence [11, 15].

The five polymorphic RC-L1 loci were genotyped, using PCR to amplify the RC-L1 5′ junctions if present and the empty site if the RC-L1 was absent, in healthy controls (minimum of 176) and SALS individuals (minimum of 173) from the MNDA UK DNA bank cohort. Association analysis of each RC-L1 locus did not demonstrate any significant association with SALS (insertion allele frequencies and p values are reported in Table 2). To extend this analysis further and increase the number of individuals genotyped without the need for PCR, tagging SNPs in the genomic region flanking the site of the L1 insertion were identified for each of the five polymorphic RC-L1 insertions listed in Table 1. Both the SNP and RC-L1 genotypes were available in a minimum 289 individuals and LD was calculated between the RC-L1 and their respective SNPs. All r^2^ values were greater than 0.90 showing strong LD between the RC-L1s and their proxy SNPs and specific values are shown for each of the five polymorphic RC-L1s listed in Table 2. This enabled the presence/absence of each RC-L1 in the UK samples from Project MinE to be determined from the genotype of their proxy SNP (Table 2). In this UK cohort there was no association of any of the five individual polymorphic RC-L1 genotypes with ALS using SNP genotypes as a proxy and p values are shown in Table 2.

**Table 2:** Allele frequencies of polymorphic RC-L1s are similar using PCR or tagging SNPs to determine L1 genotype

L1 (Tagging SNP)	IAF of L1 based on PCR		p value based on PCR genotypes	r^2^,D’	IAF of L1 based on SNP		p value based on SNP genotypes
L1_chr2_q24.1 (rs7594648)	Controls (243)	0.30	0.67	0.95,0.98	Controls (386)	0.32	0.38
	SALS (220)	0.29			SALS (1331)	0.30	
L1_chr6_q24.1 (rs1150602)	Controls (494)	0.14	0.32	0.90,1.00	Controls (340)	0.17	0.36
	SALS (445)	0.16			SALS (1178)	0.18	
L1_chr6_p22.3 (rs6932875)	Controls (180)	0.14	0.39	0.90,0.97	Controls (357)	0.16	0.99
	SALS (179)	0.12			SALS (1227)	0.15	
L1_chr8_q24.22 (rs7844570)	Controls (176)	0.45	0.66	0.99,1.00	Controls (385)	0.46	0.34
	SALS (177)	0.44			SALS (1330)	0.44	
L1_chrX_p22.2 (rs6640825)	Female controls (114)	0.57	0.31	0.94,0.99	Female controls (233)	0.59	0.56
	Female SALS (54)	0.51			Female SALS (509)	0.57	
	Male controls (64)	0.56			Male controls (139)	0.58	
	Male SALS (119)	0.55			Male SALS (740)	0.60	

Each polymorphic RC-L1 was genotyped using PCR and then expanded into the larger cohort of the UK Project MinE samples using tagging SNPs as a proxy for the L1 genotype and each method demonstrated a similar insertion allele frequency (IAF). The RC-L1s were in strong LD with their respective tagging SNPs demonstrated by the r^2^ values. There was no significant association of any of the RC-L1s with SALS by either genotyping method (chi-squared test). Number in brackets in columns IAF of L1 based on PCR and IAF of L1 based on SNP indicate the number of individuals per cohort

**Fig. 1:**
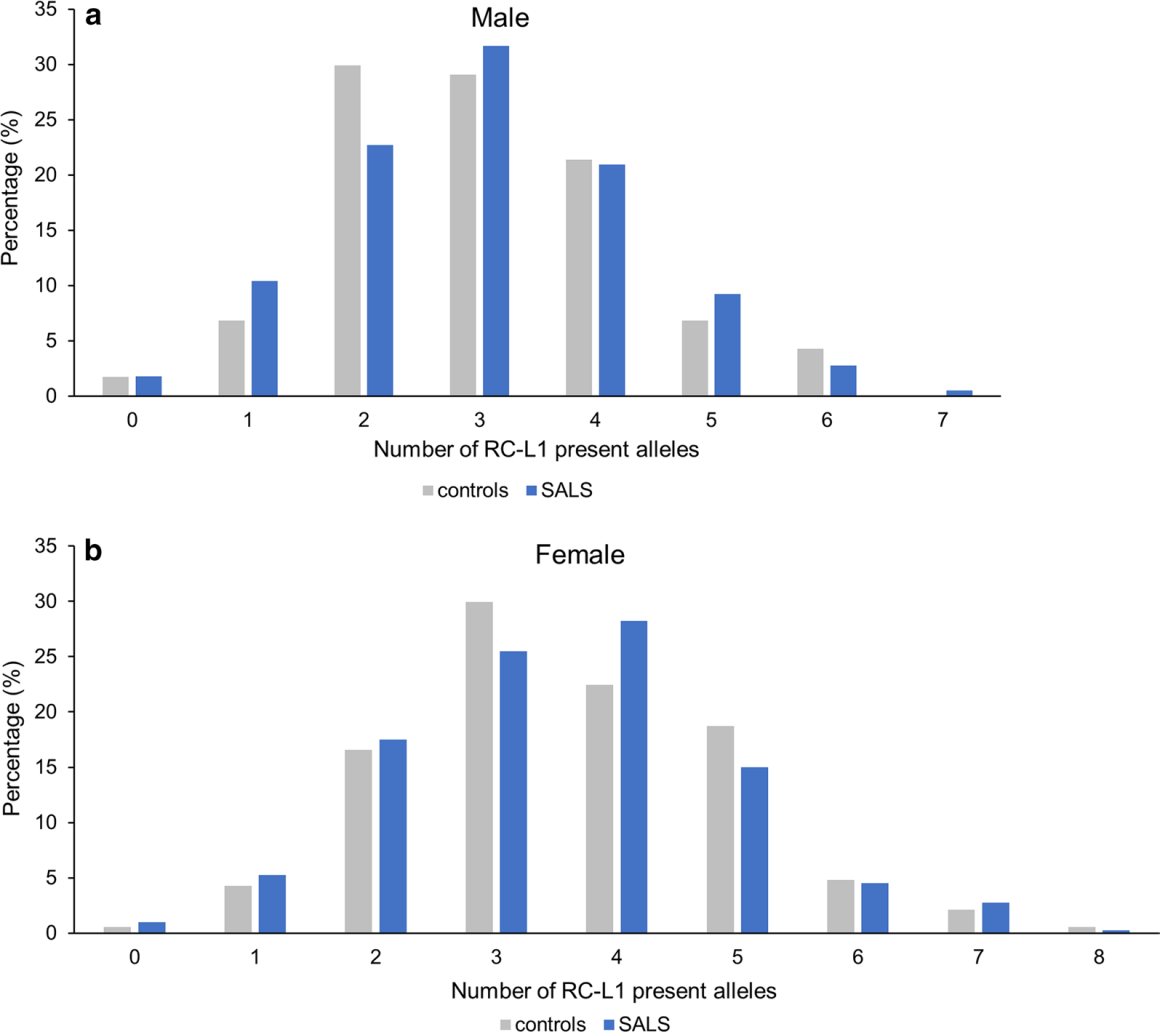
Burden of five specific RC-L1 loci in healthy controls and individuals with SALS. **a** Percentage of male individuals harbouring different numbers of specific RC-L1 present alleles of the polymorphic L1 loci listed in Table 1 was determined by using tagging SNPs as a proxy for the L1 genotype in controls and SALS patients. In males, it is possible to have up to 9 alleles with a RC-L1 present of the 5 loci genotyped. **b** Percentage of female individuals harbouring different numbers of RC-L1 present alleles of the polymorphic L1 loci listed in Table 1 was determined by using tagging SNPs as a proxy for L1 genotype in controls and SALS. In females, it is possible to have up to 10 alleles with a RC-L1 present of the 5 loci genotyped. Two-sample test for equality of proportions to compare number of RC-L1 between cases and controls. Male-Controls n = 117, SALS n = 616, Female—Controls n = 187, SALS n = 400

The risk that polymorphic RC-L1s in the germline could contribute to disease may not be due to one individual RC-L1 insertion but a combination of multiple RC-L1 elements within the genome. Due to the presence or absence of the polymorphic RC-L1 insertions, the total number of alleles across the five loci harbouring an RC-L1 insertion will vary between individuals. Therefore, for each individual, we determined the total number of alleles that contained an RC-L1 insertion at the five polymorphic loci listed in Table 1. A male could harbour between 0 and 9 and a female between 0 and 10 alleles with a RC-L1 insertion present at the five RC-L1 loci genotyped in this study. Using the genotypes of the proxy SNPs for each RC-L1 locus in the Project MinE UK cohort the total number of present alleles per individual was determined for the five polymorphic RC-L1 loci (Table 1). In males, 1.7% of healthy controls and 1.8% of the individuals with ALS did not carry any of the five RC-L1 insertions in Table 1. In male controls and people with SALS, the greatest number of RC-L1 loci (out of those listed in Table 1) per genome was 6 and 7, respectively (Fig. 1a). In females 0.53% of healthy controls and 1% of people with ALS did not carry any of the five RC-L1 insertions presented in Table 1 and the greatest number of RC-L1s at the five polymorphic loci per genome in both the controls and people with SALS was 8 (Fig. 1b). Logistic regression analysis in males and females revealed there was no association of the number of RC-L1s present at the 5 polymorphic loci analysed with disease status (males p = 0.90, females p = 0.64). For the RC-L1 loci investigated here, there was no increased burden of their presence in ALS genomes from 1320 individuals of the UK Project MinE cohort.

### Six selected RC-L1 loci exhibit lower methylation levels of the L1 promoter region in the motor cortex of familial and sporadic ALS brains compared to control brains

**Fig. 2:**
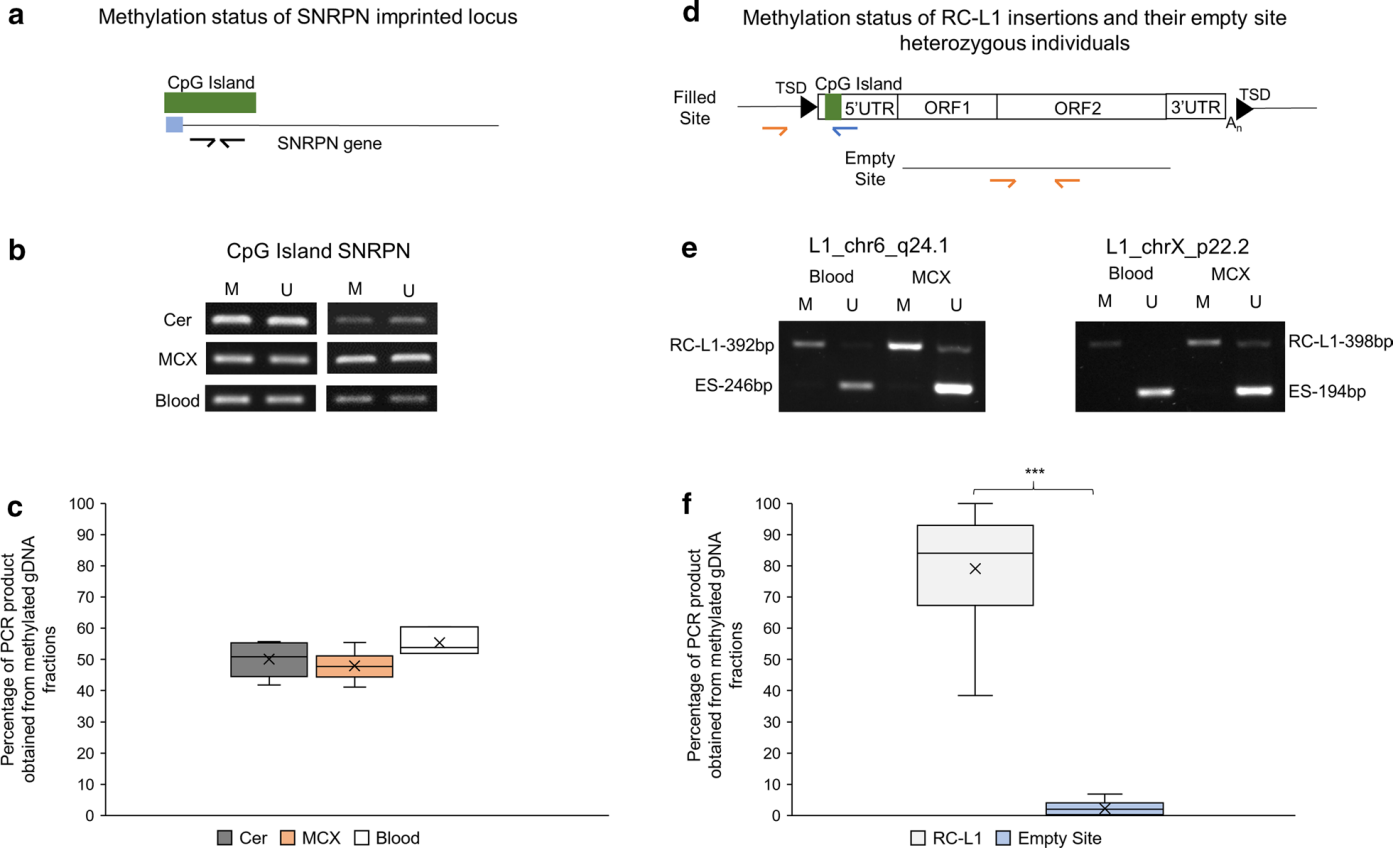
Analysis of methylation status of imprinted *SNRPN* locus and heterozygous RC-L1 insertions. **a** Schematic showing location of binding sites of primers in the *SNRPN* locus used to PCR amplify its CpG island as an internal control for the successful enrichment of methylated and unmethylated gDNA fractions. Blue box –exon. **b** Example gel image of PCR product (263 bp) amplified from *SNRPN* CpG island in methylated and unmethylated fractions of DNA from all three tissues. (*Cer* cerebellum, *MCX* motor cortex, *M* methylated fraction of DNA, *U* unmethylated fraction of DNA). **c** Box-Whisker plot demonstrating methylation status of the CpG island of the imprinted *SNRPN* locus in cerebellum, motor cortex and blood of 9 individuals. Methylation status was determined by comparing the band intensity of the PCR product in the methylated and unmethylated fractions of gDNA. The average methylation of the CpG island of the *SNRPN* locus in cerebellum, motor cortex and blood comprises 55%, 48% and 55%, respectively. Cerebellum, n = 6 (controls = 2, FALS = 2, SALS = 2); MCX, n = 9 (controls = 2, FALS = 2, SALS = 5); blood, n = 3 (SALS = 3). **d** Schematic showing the position of binding sites of primers used to amplify the genomic 5′ junction of the selected full length RC-L1 loci when present (filled site) and the empty site when the respective RC-L1 is absent. The L1 5′UTR-specifc primer binds position 223–206 of the L1.3 reference sequence TSD, target site duplication; UTR, untranslated region; ORF1 and ORF2, open reading frame 1 and 2; A_n_, polyA stretch; **e** PCR analysis of 5′ junctions of RC-L1 loci L1-chr6-q24.1 and L1-chrX-p22.2 and the empty sites of heterozygous carriers in the methylated and unmethylated gDNA fractions from blood and motor cortex of same individual. **f** The 5′ junction of the RC-L1s is enriched in the methylated fraction of DNA, while the empty sites (absent allele) lack any meaningful methylation in heterozygous carriers. Average methylation status of 5′ junctions RC-L1 loci and empty sites comprises 79% and 2%, respectively (14 heterozygous RC-L1 loci). Two-tailed t-test *** p < 0.001 X = mean

gDNA samples isolated from motor cortex, cerebellum and blood from controls and people with sporadic or familial ALS (a total of nine individuals) were enriched for methylated gDNA fragments by pulling down methylated gDNA using MBD protein and thereby separating methylated from unmethylated gDNA fractions. The specific gDNA targets of interest were then amplified from both fractions by PCR in order to determine if the region of interest was enriched either in the methylated or unmethylated gDNA fraction. As an internal control, the CpG island of the imprinted locus small nuclear ribonucleoprotein polypeptide *N* (*SNRPN*) was amplified in each gDNA sample to ensure successful isolation of methylated and unmethylated DNA fractions as only one of the two *SNRPN* alleles is methylated. Amplifying this CpG island also demonstrated on average an equal representation of the methylated and unmethylated allele in the respective gDNA fractions. The average relative methylation of the CpG island of the *SNRPN* locus in cerebellum, motor cortex and blood comprised of 50%, 48% and 55%, respectively (Fig. 2a–c).

Six out of the seven RC-L1s shortlisted in Table 1 (L1_chr1_p12, L1_chr22_q12.1, L1_chr6_q24.1, L1_chrX_p22.2, L1_chr6_p22.3 and L1_chr8_q24.22) were analysed for their relative levels in methylated and unmethylated gDNA fractions of DNA. The RC-L1 locus L1_chr2.q24.1 had to be excluded from our analysis, because it was not possible to generate the RC-L1 locus specific 985-bp PCR product in the sheared gDNA used as input in MBD assay. People heterozygous for the presence of a particular RC-L1 insertion allowed for the simultaneous comparison of this region of gDNA with and without the RC-L1 present to determine the effect of the 5′ UTR of the L1 on methylation status. At the 14 heterozygous RC-L1 loci analysed, the allele lacking the RC-L1 insertion had a significantly lower level of methylation compared to the allele harbouring the RC-L1 insertion (average relative methylation of empty site and RC-L1 5′end equals 2% and 79%, respectively; p < 0.001) across all types of tissue (cerebellum, motor cortex and blood) (Fig. 2d–f). Figure 2e shows presence/absence PCR analyses of the genomic RC-L1 loci L1_chr6_q24.1 and L1_chrX_p22.2 with the empty site being enriched in the unmethylated gDNA fractions from both blood and motor cortex relative to the 5′ junctions of RC-L1 loci that are enriched in the methylated fractions. This comparison confirmed that the differential methylation is associated with the insertion of the RC-L1, which would be expected due to the introduction of the L1 CpG island, rather than the nature of the flanking gDNA.

**Fig. 3:**
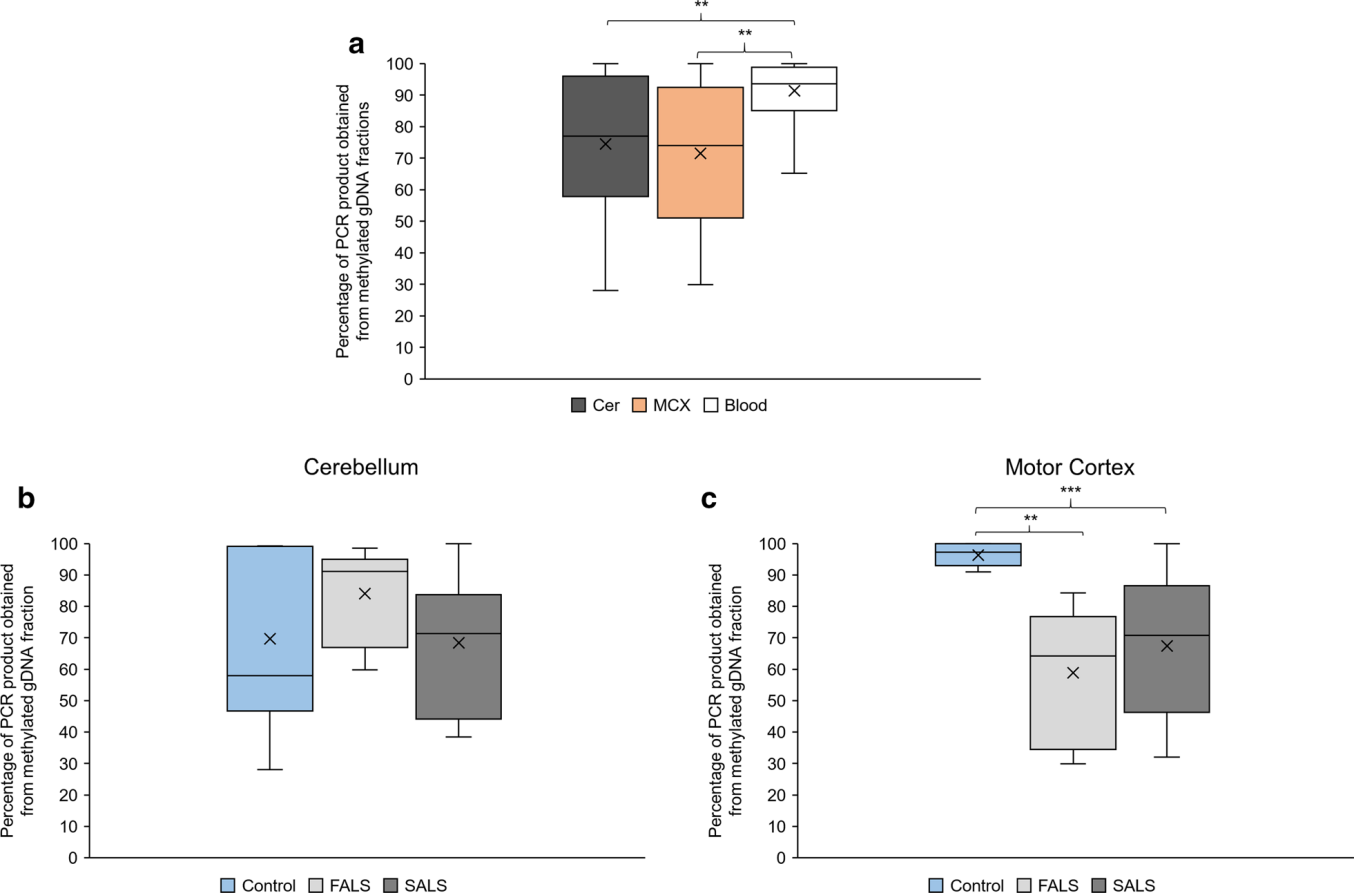
Comparison of the methylation of six selected RC-L1 loci by tissue and affected status. **a** Box-Whisker plots indicate the methylation status of the 5′ junctions of six RC-L1 loci (L1_chr1_p12, L1_chr22_q12.1, L1_chr6_q24.1, L1_chrX_p22.2, L1_chr6_p22.3 and L1_chr8_q24.22; see Table 1) in the cerebellum (Cer), motor cortex (MCX) and blood. The average methylation status of 5′ junctions of the RC-L1 loci in cerebellum, motor cortex and blood constituted 75%, 72%, and 91%, respectively. Number of RC-L1 5′ junctions analysed – Cer n = 22, MCX n = 33, blood n = 11 across 9 individuals (controls = 2, FALS = 2, SALS = 5). **b** Box-Whisker plots presenting the methylation status of the 5′ junctions of the six RC-L1 loci (see above) in the cerebellum of control individuals (n = 2), and FALS (n = 2) and SALS (n = 2) patients. The average methylation status of the 5′ junctions of the RC-L1 loci in control individuals, FALS and SALS patients amounted to 75%, 82% and 68%, respectively. 22 RC-L1 5′ junctions were analysed in the cerebella (controls n = 7, FALS n = 8, SALS n = 7). **c** Box-Whisker Plots presenting the methylation status of the 5′ junctions of the six RC-L1 loci (see above) in the motor cortex of control individuals (n = 2), and FALS (n = 2) and SALS (n = 5) patients. The average methylation status of the 5′ junctions of the RC-L1 loci in control individuals, FALS and SALS patients amounted to 96%, 63% and 70%, respectively. 33 RC-L1 5′ junctions were analysed in the motor cortices (controls n = 7, FALS n = 8, SALS n = 18). Two-tailed t-test ** p < 0.01, *** p < 0.001 X = mean

To determine if the methylation state of the RC-L1s was tissue dependent, we compared the percentage of the RC-L1 5′ junction in the methylated fraction of gDNA from the cerebellum, motor cortex and blood of all individuals (controls, FALS and SALS). While 5′ junctions of RC-L1 loci are on average methylated to the same degree in cerebellum and motor cortex, there is a significant difference in the methylation status between the highly methylated RC-L1 5′ junction in blood relative to the less efficiently methylated L1 promoter regions in neuronal tissues tested (cerebellum—75%, motor cortex—72% and blood—91%) (Fig. 3a and Additional file 3a). When comparing the average methylation status of 5′ junctions of the six selected RC-L1 loci in the cerebellum of 2 control brains with those of 2 FALS and 2 SALS brains (Fig. 3b) there was no significant difference (controls—75%, FALS—82%, SALS—68%). Although a higher proportion of RC-L1 5′ junctions was methylated in people with FALS than in people with SALS or controls. However, in the motor cortex of people with FALS and SALS, the average percentage of the RC-L1 5′ junction in the methylated fraction was significantly lower than compared to the controls (controls—96%, FALS—63%, SALS—70%) (Fig. 3c and Additional file 3b). In the controls 91–100% of the PCR products obtained from 5′ junctions of the selected RC-L1 loci were derived from the methylated fraction depending on the locus, while contrarily 30–84% in FALS and 32–100% in SALS were derived from the methylated fraction. This indicates that in the motor cortex of people with FALS and SALS, the 5′ junctions of the selected RC-L1 loci exhibit significantly lower levels of CpG methylation on average compared to healthy controls (Fig. 3c). Therefore, we set up the hypothesis that the expression of these RC-L1 loci may be increased in the motor cortex of people with SALS or FALS. To confirm this hypothesis, future work has to investigate both expression of functional L1 loci in the motor cortex of people with FALS and SALS relative to healthy controls and potential L1 retrotransposition events that would be expected to occur as a consequence of transcriptional upregulation of RC-L1 loci in this specific tissue. Due to the limited amounts of available patient material, it was not possible to include such experiments in our presented study.

## Discussion 

The human genome harbours two sets of RC-L1 loci. There is one set that all individual genomes have in common, and the other set whose members are polymorphic for their presence or absence. Therefore, numbers, genomic locations and mutagenic potential of RC-L1s differ between individuals. Our study focussed on seven highly active RC-L1 loci (including five polymorphic L1 elements and two L1 insertions fixed in the reference genome) of the 80 functional reference L1 insertions [11] and ~ 100 functional non-reference L1 loci were reported to date [14, 15, 17, 48]. The five polymorphic RC-L1 loci were genotyped by PCR amplification initially (between 351 and 939 individuals involved depending on the RC-L1 locus analysed) and tagging SNPs were used to increase the number of individuals analysed (between 1518 and 1717 individuals depending on the SNPs passing quality control) who were taken from the UK MNDA DNA bank cohort and the UK samples from Project MinE. We determined that the five polymorphic RC-L1s were not associated with SALS individually and an increasing burden of these five specific RC-L1 loci did not affect the likelihood of having the disease (Table 2 and Fig. 1).

An essential prerequisite for L1 mobilisation is the expression of functional L1 mRNA and the L1-encoded protein machinery including ORF1p and ORF2p from genomic RC-L1 elements. L1 expression is regulated by methylation of a CpG island located in the L1 5′UTR between pos. 30 and pos. 450 of an intact L1 element and this is mediated by a highly conserved Yin Yang 1 transcription factor binding site [19, 20, 49, 50]. We assessed the methylation status of the CpG-island of the 5′ UTR of six specific RC-L1 loci in gDNA from motor cortex and matching cerebellum or blood isolated from those individuals with or without disease. On average, the level of methylation was significantly lower in the two brain regions analysed when compared to the blood (Fig. 3a), which is consistent with the established fact that L1s have the ability to retrotranspose in neuronal cells. Our PCR analyses identified a significantly higher level of methylation of the promoter region of the listed six RC-L1 loci in the motor cortex (the region of the brain primarily affected by ALS) of control brains compared to those from people with either sporadic or familial ALS (Fig. 3c). However, this study was limited to a small number of available samples from two controls, two people with FALS and five with SALS therefore further work would be needed to establish if this reduced methylation is a reoccurring feature of disease or natural variation in the level of methylation of these elements across different people. Reduced methylation of RC-L1 promoters has been shown to lead to an increase in the expression of functional L1 gene products and L1 retrotransposition [20, 51, 52]. Consistently, patients with Rett Syndrome carrying mutations in MeCP2, which establishes repression by DNA methylation through binding to the methylated gDNA, have increased susceptibility for L1 retrotransposition [53]. Activation of L1 retrotransposons confer genomic and cellular instability as L1 insertions can disrupt coding regions and modify epigenetic and post-transcriptional regulation of gene expression. Destabilisation of the genome can be caused by an increase in endogenous L1 retrotransposition frequency and by L1-encoded endonucleolytic activity of ORF2p, which can cause genomic DNA damage that could also negatively affect health and survival of the cell [4, 6, 54]. The high metabolic activity of neurons and their inability to use replication-coupled DNA repair mechanism makes them vulnerable to DNA damage, which was reported to occur in neurodegenerative disorders including ALS [55, 56]. In addition, a *Drosophila* model of ALS demonstrated the de-repression of transposable elements (including members of the L1 superfamily) that were involved in DNA damage-induced cell death of neurons [37]. It has also been suggested that the expression of functional L1 proteins could increase the L1 DNA content of a cell without retrotransposition occurring due to the extrachromosomal accumulation of L1 cDNA that had not integrated into the nuclear genome due to failed or incomplete target primed reverse transcription [29]. This could potentially have damaging effects by triggering an immune response or tying up host factors involved in regulating cellular processes [29, 57–60]. A recent study of ALS-FTD brains, using a qPCR assay that did not distinguish if changes in L1 copy number is due to successful retrotransposition or any other mechanism raising the number of L1 copies in gDNA preparations, showed an increase in the L1 copy numbers in TDP-43 negative nuclei [41]. Other neurological diseases, including Rett Syndrome and ataxia telangiectasia, have also been associated with elevated L1 DNA content [53, 61].

ALS is a complex disorder involving both genetic and environmental risk factors for developing the disease with multiple steps in the molecular processes that result in ALS [62]. Our study of a small number of highly active RC-L1s did not identify an association of their genotype with ALS. However, there are approximately 165 potential alternative RC-L1 loci across the human genome and therefore further studies will be required to fully explore their potential to be part of the genetic landscape that predisposes an individual to ALS. Applying bioinformatics tools such as Mobile Element Locator Tool [15], for genotyping both reference and non-reference L1 insertions using whole genome sequencing data would enable a genome wide analysis of all known RC-L1 loci. This would allow for a more comprehensive analysis of the potential contribution of RC-L1 loci to the genetic risk of ALS in future studies. However, our pilot study did demonstrate that the methylation of CpG-islands of the 5′UTRs of the selected RC-L1 loci is reduced in the motor cortex of those people with ALS relative to healthy individuals in the small number of samples analysed here. Changes to RC-L1 regulation could be involved in downstream molecular events of the disease process influencing cellular function through various mechanisms that include direct effects of the RC-L1 mRNA and proteins such as mobilisation or the through their modulation of expression of neighbouring genes. Our study has highlighted RC-L1s as important genetic elements for further investigation in ALS. Further studies to expand on these findings to help decipher the role of the elements should include addressing the question of the level of mobilisation, a genome wide approach to their variation and methylation status, determining mRNA expression levels from RC-L1s and the effect of the presence or absence of these insertions on gene expression.

## Supplementary information

**Additional file 1.** Details of samples used in methylation analysis of RC-L1s.

**Additional file 2.** Primer sequences used to amplify empty site and 5′ junction of RC-L1 loci and *SNRPN* locus.

**Additional file 3.** Example gel images of 5’ junction amplification of RC-L1s in unmethylated and methylated fraction of gDNA.

## Data Availability

The datasets used and/or analysed during the current study are available from the corresponding author on reasonable request.

## Abbreviations

ALS: Amyotrophic lateral sclerosis
FALS: Familial amyotrophic lateral sclerosis
FTD: Frontotemporal dementia
LINE-1/L1: Long interspersed element-1
LD: Linkage disequilibrium
MBD: Methyl binding domain
ORF: Open reading frame
Non-LTR: Non long terminal repeat
RC: Retrotransposition competent
SALS: Sporadic amyotrophic lateral sclerosis
TSD: Target site duplication
UTR: Untranslated region
