# Supplementary material for: Frequency and methylation status of selected retrotransposition competent L1 loci in amyotrophic lateral sclerosis
Source: Mol Brain. 2020 Nov 13;13:154. doi: 10.1186/s13041-020-00694-2 (PMC7666467; doi:10.1186/s13041-020-00694-2)
Supplement: Supplementary file 1 — Additional file 1. Details of samples used in methylation analysis of RC-L1s. [file 13041_2020_694_MOESM1_ESM.docx]

| Brain bank | Age | Gender | Phenotype | Mutation | Tissue |
| --- | --- | --- | --- | --- | --- |
| LNDBB | 99 | Female | Control | NA | Cer, MCX |
| LNDBB | 66 | male | Control | NA | Cer, MCX |
| LNDBB | 46 | female | Familial ALS | SOD1 p.D101G | Cer, MCX |
| LNDBB | 57 | male | Familial ALS | TARDBP p.M337V | Cer, MCX |
| LNDBB | 75 | female | Sporadic ALS | unknown | Cer, MCX |
| LNDBB | 76 | female | Sporadic ALS | unknown | Cer, MCX |
| SBTB | unknown | unknown | Sporadic ALS | unknown | Blood, MCX |
| SBTB | unknown | unknown | Sporadic ALS | unknown | Blood, MCX |
| SBTB | unknown | unknown | Sporadic ALS | unknown | Blood, MCX |

Additional file 3: Details of samples used in methylation analysis of RC-L1s. LNDBB - London Neurodegenerative Diseases Brain Bank, SBTB – Sheffield brain tissue bank, Cer – cerebellum, MCX – motor cortex.
