# Supplementary figures and images for: Frequency and methylation status of selected retrotransposition competent L1 loci in amyotrophic lateral sclerosis
Source: Mol Brain. 2020 Nov 13;13:154. doi: 10.1186/s13041-020-00694-2 (PMC7666467; doi:10.1186/s13041-020-00694-2)

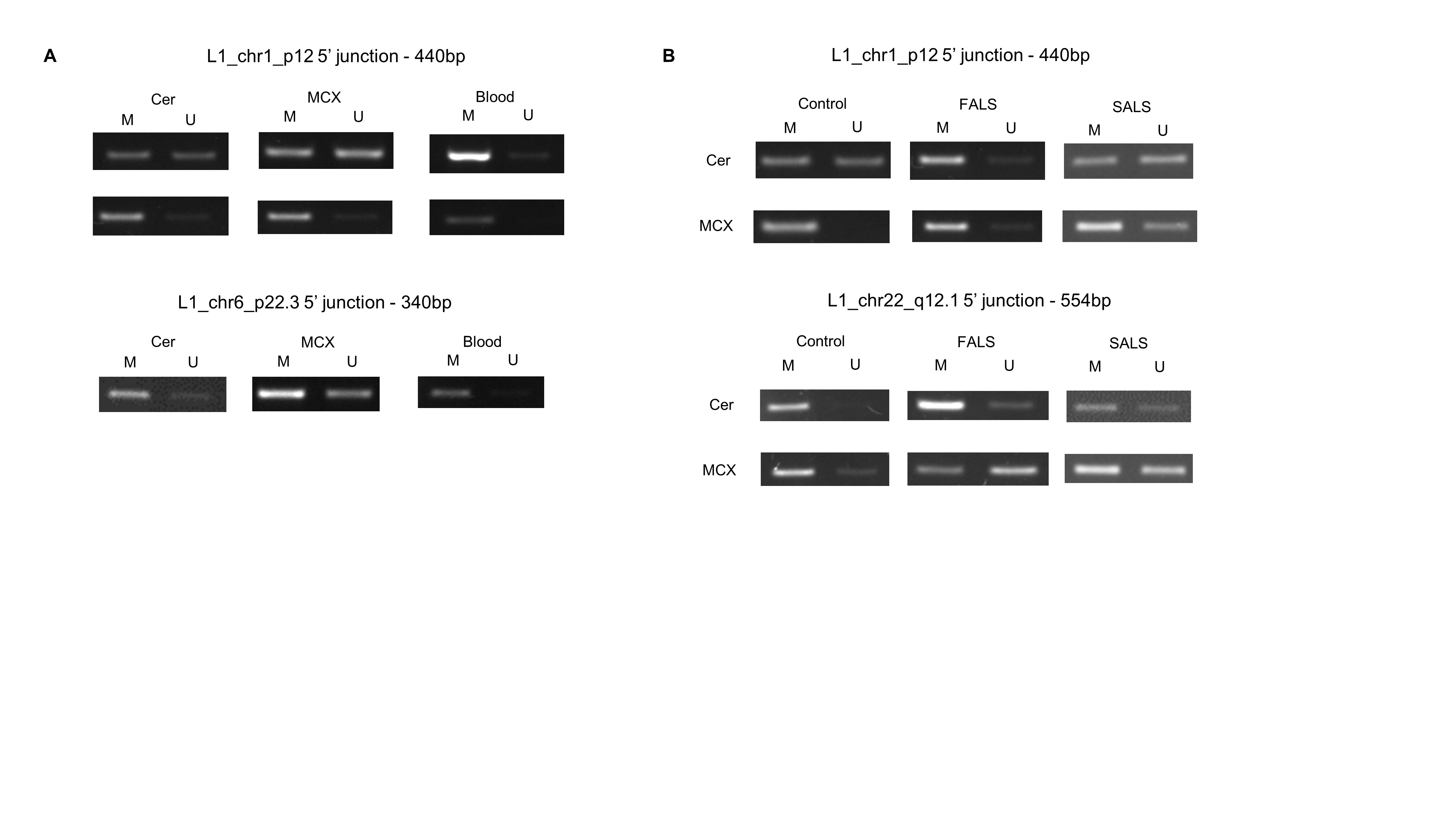

Supplement: Supplementary file 3 — Additional file 3. Example gel images of 5’ junction amplification of RC-L1s in unmethylated and methylated fraction of gDNA. [file 13041_2020_694_MOESM3_ESM.tif]
